# Differential Mammary Gland Development in FVB and C57Bl/6 Mice: Implications for Breast Cancer Research

**DOI:** 10.3390/nu3110929

**Published:** 2011-10-25

**Authors:** Mira B. MacLennan, Breanne M. Anderson, David W.L. Ma

**Affiliations:** Department of Human Health & Nutritional Sciences, University of Guelph, Guelph, ON, N1G 2W1, Canada; Email: mmaclenn@uoguelph.ca (M.B.M.); banderso@uoguelph.ca (B.M.A.)

**Keywords:** mammary gland, terminal end buds (TEB), FVB, C57Bl/6, breast cancer

## Abstract

A growing body of research suggests a linkage between pubertal mammary gland development and environmental factors such as diet as modifiers of long term breast cancer risk. Much of this research is dependent upon mouse models, which may vary between studies. However, effects may be strain dependent and further modified by diet, which has not been previously examined. Therefore, the objective of the present study was to determine whether mammary gland development differs between FVB and C57Bl/6 strains on diets containing either *n*-6 or *n*-3 polyunsaturated fats. Developmental measures related to onset of puberty and mammary gland development differed between strains. Mice fed the *n*-3 polyunsaturated fatty acids (PUFA) diet were shown to have lower numbers of terminal end buds, a marker of mammary gland development. This study helps to further clarify differences in development and dietary response between FVB and C57Bl/6 mice in order to more appropriately relate mammary gland research to human populations.

## 1. Introduction

The use of mouse models is common in the field of breast cancer research, as mammary gland (MG) development is comparable between species and studies are more easily executed in rodent models. However, it is becoming increasingly evident that there are important developmental differences between mouse strains used. In studies on obesity and diabetes, varying responses of insulin resistance have been observed to fluctuate depending on background strain [1]. When looking at development of specific tissues such as in the MG, exposure to hormones such as progesterone and estrogen elicit drastically different responses depending on the strain [2,3]. Similarly, consuming a high fat diet can cause opposing effects on mammary epithelial cell proliferation depending on the background strain of the mouse model [4]. Such differences need to be considered when extrapolating findings to human populations, since findings may be strain dependent.

MG development, and its relation to breast cancer development, is of interest for cancer prevention research. Much of the body of work in this field has used rodents because MG development closely resembles that of the human breast [5,6]. The MG begins as a blank fat pad, with a primary duct at the nipple. This primary duct branches posteriorly throughout the fat pad and progressively infiltrates the MG throughout life. These ducts are led by zones of high cell proliferation, termed terminal ductal lobular units (TDLU) and eventually differentiate into lobulo-alveolar units (LAU) later in life when full maturation of the MG has been reached, typically due to full-term pregnancy and lactation. The ductal system in rodents experiences a similar pattern of growth. The TDLU and LAU in humans have equivalencies in rodents called terminal end buds (TEB) and alveolar buds (AB), respectively [5,6]. TEB have been identified as sites of tumour initiation and hence a point of interest linking MG development to breast cancer risk.

Prevention research has focused on the effect of dietary fat on MG development [7]. Western diets typically consist of an abundance of *n*-6 polyunsaturated fatty acids (*n*-6 PUFA) and little *n*-3 PUFA, whereas typical Asian diets consist of high levels of *n*-3 PUFA and have been correlated with a lower incidence of breast cancer [8]. Rodent studies suggest that *n*-6 PUFA and *n*-3 PUFA have opposing effects on TEB formation, with *n*-3 PUFA tending to reduce the number of TEB that form throughout life [9,10]. The interaction between diet, strain and timing of exposure remains poorly understood and therefore extrapolation of results from animal-based studies need to be cautiously interpreted. Just as there is diversity within the human population, so is there diversity within other species and mice are no exception. Currently, there are a limited number of studies comparing strain dependent differences in development [1,2,3,4,11,12]. Of these, a few have looked at MG development in BALB/c and C57Bl/6 mice [2,3,4] and mammary tumour development in FVB and C57Bl/6 mice [11], however none have looked at the role of different dietary fatty acids in this context. Given the increasing interest in breast cancer prevention through diet [7], it is important to consider potential strain dependent outcomes that modify dietary effects, which may affect our interpretations of results in this field of research. Therefore, the present study examines how effects of dietary *n*-6 and *n*-3 PUFA on MG development are differentially modified in two common mouse strains, FVB and C57Bl/6.

## 2. Material and Methods

Mice: Male breeders were taken from FVB and C57Bl/6 colonies maintained in the Ma lab. FVB and C57Bl/6 female breeders were ordered from Charles River. All mice used were wildtype and housed in a temperature and humidity controlled facility on 12-12 h light-dark cycle. Water and food were provided ad libitum. All experimental procedures were approved by the institutional animal care committee (University of Guelph).

Diet: Two experimental diets were used with varying fatty acid compositions; one contained 10% safflower oil wt/wt (*n*-6 PUFA diet), and the other contained 7% safflower oil wt/wt and 3% menhaden oil wt/wt (*n*-3 PUFA diet). Mice were maintained on their specified diet throughout life, including in harem and litter cages, providing a consistent diet from conception to termination.

Puberty Onset: Date of vaginal opening was used as a marker of puberty onset in female mice. Mice were checked daily [13].

Euthanasia: Mice were euthanized by CO_2_ asphyxiation at specified timepoints of 3 weeks (pre-puberty) or 6 weeks (puberty) of age, when TEB typically reach their maximum. On the day of euthanasia, vaginal smears were taken from mice to determine stage of estrous cycle. In order to control for cyclical effects of diestrus elicited on the MG [14], mice in diestrus were not taken that day and kept for a later timepoint. Mice in any other stage of estrous were taken as scheduled. Mice not in diestrus were weighed, euthanized and mammary glands were extracted for further analysis.

Wholemounting: Left 4th MG were fixed in 10% formaldehyde for 48 h, placed in acetone for two days (refreshed daily), rehydrated and stained in carmine alum overnight, then dehydrated and cleared using xylenes, and sealed in methyl salicyclate. MG were then scored blindly by two independent investigators for terminal end buds (TEB) using a Zeiss stereoscope. Fourth MG were used for wholemounting as is common practice when studying murine MG [6,15,16].

Statistics: Statistical analyses were conducted using SASv 9.1. A two-way ANOVA was used to analyze data on strain and diet, however since there were no significant interactions between strain and diet, main effects of strain and diet at each timepoint were analyzed separately by Student *T*-tests. Values were considered significant when *p* < 0.05.

## 3. Results

### 3.1. FVB Mice

**Figure 1:**
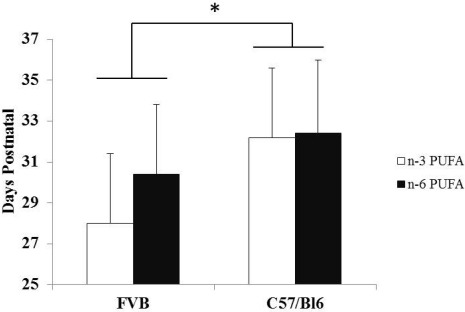
Puberty Onset in FVB and C57Bl/6 mice by Diet. White bars indicate mice on *n*-3 PUFA diet (FVB *n* = 13; C57Bl/6 *n* = 18) and black bars indicate mice on *n*-6 PUFA diet (FVB *n* = 15; C57Bl/6 *n* = 7). * Overall, a significant (*p* < 0.05) main effect of strain was observed by analysis of variance showing that pubertal onset was earlier in FVB than C57Bl/6 mice. Values are in mean ± SD.

Puberty Onset-Although not significant (*p* = 0.07), vaginal opening in FVB mice maintained on an *n*-3 PUFA diet was observed to occur at an earlier age, 28.0 ± 3.4 days (*n* = 13), than the *n*-6 PUFA diet group, 30.4 ± 3.4 days (*n* = 15) (Figure 1).

TEB Enumeration-MG taken from FVB mice at 3 weeks of age (pre-puberty) did not differ in TEB formation between *n*-6 PUFA (3.4 ± 4.2 TEB; *n* = 17) and *n*-3 PUFA (4.1 ± 2.3 TEB; *n* = 12) diet groups. However, at 6 weeks of age (puberty) there was a significant difference (*p* < 0.05) in TEB formation between diet groups, with *n*-6 PUFA having a higher number of TEB (18.7 ± 4.0 TEB; *n* = 15) than MG from mice consuming an *n*-3 PUFA diet throughout life (14.9 ± 3.3 TEB; *n* = 13) (Table 1).

**Table 1:** Terminal end buds (TEB) Development in FVB and C57Bl/6 Mice. TEB enumeration was conducted at 3 and 6 weeks of age. A significant (*p* < 0.05) diet effect was observed in both strains of mice at 6 weeks of age. Within a row, * indicates significant difference (*p* < 0.05) between diets within a strain. Values are in mean ± SD (*n*).

		*n*-6 PUFA	*n*-3 PUFA
6 weeks	FVB	18.7 ± 4.0 (15)	* 14.9 ± 3.3 (13)
	C57Bl/6	19.9 ± 8.5 (7)	* 11.3 ± 8.7 (18)

### 3.2. C57Bl/6 Mice

Puberty Onset-Similar to the FVB mice, there was no significant difference in puberty onset. Vaginal opening in C57Bl/6 mice maintained on *n*-3 PUFA diet occurred at 32.2 ± 3.4 days (*n* = 18) compared to 32.4 ± 3.6 days when on an *n*-6 PUFA diet (*n* = 7).

TEB enumeration-MG taken from C57Bl/6 at 3 weeks of age (pre-puberty) exhibited fewer TEB in mice fed *n*-6 PUFA (0.1 ± 0.22 TEB; *n* = 5) than in mice fed *n*-3 PUFA (4.4 ± 4.85 TEB; *n* = 16) diet groups, but the difference was not significant (*p* = 0.07). At 6 weeks of age (puberty) however, a significant difference was found in C57Bl/6 TEB formation between diet groups (*p* < 0.05), with *n*-6 PUFA having more TEB (19.9 ± 8.5 TEB; *n* = 7) compared to those maintained on an *n*-3 PUFA diet (11.3 ± 8.7 TEB; *n* = 18) (Table 1).

### 3.3. Strain Differences between FVB and C57Bl/6 Mice

Puberty Onset-Overall, there was a significant strain effect on vaginal opening (*p* < 0.01), which occurred earlier in FVB than C57Bl/6 (Figure 1). Irrespective of diet, vaginal opening occurred on average at 29.3 ± 3.5 days of age in FVB mice (*n* = 28) and 32.3 ± 3.4 days of age in C57Bl/6 mice (*n* = 25).

**Figure 2:**
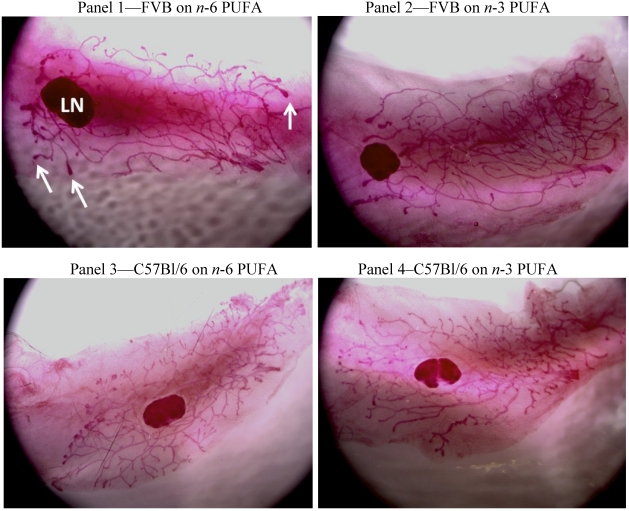
Representative stereoscopic wholemount images (20×) of the left fourth mammary gland (MG) at 6 weeks of age, from FVB and C57Bl/6 mice fed *n*-6 or *n*-3 PUFA. FVB on *n*-6 PUFA (Panel 1), FVB on *n*-3 PUFA (Panel 2), C57Bl/6 on *n*-6 PUFA (Panel 3) and C57Bl/6 on *n*-3 PUFA (Panel 4). Arrows show representative terminal end buds (TEB) enumerated. Lymph node (LN).\

TEB Enumeration-No significant differences were found by diet or by strain in the number of TEB in MG collected from mice at 3 weeks of age (pre-puberty). However, in both strains, there was a significant difference found at 6 weeks of age (puberty) between diet groups (*p* < 0.01) with fewer TEB exhibited in MG from mice maintained on an *n*-3 PUFA diet (Table 1). See Figure 2 for representative images of MG taken at 6 weeks of age from each group.

## 4. Discussion

Vaginal opening is an indicator of puberty onset in mice, and there is evidence linking an earlier puberty onset with an increase in breast cancer risk due to the increase in estrogen exposure throughout life [10,13,17,18,19]. Thus, if the onset of puberty is delayed, the risk of breast cancer could potentially be reduced by reducing the overall exposure of estrogen over the lifetime, as has been found in both rodent [10,13] and human studies [17,18,19]. In this study, FVB mice reached puberty significantly earlier than C57Bl/6 mice (Figure 1). Timing of puberty onset has long term health implications, more specifically with early puberty onset being linked to increasing breast cancer risk later in life [20,21,22].

The effect of diet on the number of TEB was observed in both FVB and C57Bl/6 strains. At 6 weeks of age, the number of TEB in both strains was lower in the mice maintained on the *n*-3 PUFA diet than in mice maintained on the *n*-6 PUFA diet. TEB have been identified as the sites for tumour initiation and therefore these findings potentially lend support to a long term protective effect of *n*-3 PUFA through modulation of MG development. These findings are consistent with studies that have found exposure to *n*-3 PUFA linked to decreased breast cancer risk in humans [23].

It is unclear as to the specific mechanisms by which *n*-3 PUFA affects the numbers of TEB, but likely involves altered cell proliferation and apoptosis. At the cellular level, *n*-3 PUFA are known to have anti-proliferative properties compared to *n*-6 PUFA, and since TEB are zones of high cell proliferation, providing the cellular environment enriched with *n*-3 PUFA has been found to reduce levels of proliferation and increase apoptosis in early TEB formation, thus reducing overall TEB numbers [7,24,25,26]. The findings of the present study support the anti-proliferative effects of *n*-3 PUFA relative to *n*-6 PUFA reported by other studies [10,13,27]. It is evident that there are also strain differences. This is consistent with other studies showing that C57Bl/6 mice had strain specific increases in adiposity and exhibited stunted mammary growth when consuming a high fat diet during pubertal development [2,3,4]. Although it is desirable to consistently use the same strain, it may be argued that studies should include several strains if possible in order to assess the dynamic range of a given response or outcome, which may be useful when generalizing potential effects to humans.

## 5. Implications

The findings from this study highlights the potential need to consider the strain of the mouse model used when interpreting results, as different mouse strains may exhibit unique characteristics in their developmental patterns. Taking such details into consideration may provide a more accurate understanding of mammary gland development in mice and how it may best simulate human development. This has potential implications for research on mammary gland development as it relates to our furthered understanding of breast cancer prevention and treatment.
